# Engineered microorganisms unlock a new era of diterpenoid biomanufacturing: recent advances and future perspectives

**DOI:** 10.1186/s40643-026-01094-5

**Published:** 2026-07-09

**Authors:** Yuting Han, Yuanhe Luo, Kaifeng Wang, Lu Lin, Jian Gao, Mei-Li Sun, Xiao-Jun Ji

**Affiliations:** 1 State Key Laboratory of Materials-Oriented Chemical Engineering, College of Biotechnology and Pharmaceutical Engineering, Nanjing Tech University, Nanjing, 211816 People’s Republic of China; 2 School of Marine and Bioengineering, Yancheng Institute of Technology, Yancheng, 224051 People’s Republic of China

**Keywords:** Diterpenoids, Synthetic biology, Biomanufacturing, Microbial cell factories

## Abstract

Diterpenoids are natural compounds composed of four isoprene units. They possess diverse biological activities and widespread applications in the cosmetics, food additives and pharmaceutical. With the rapid advancement of synthetic biology, the biomanufacturing of diterpenoids via microbial metabolism has witnessed substantial advancements. Microbial chassis such as *Escherichia coli*, *Saccharomyces cerevisiae*, *Yarrowia lipolytica* and *Rhodosporidium toruloides*, have been successfully engineered to enable efficient biosynthesis of these compounds, thereby demonstrating substantial potential for industrial-scale applications. In this review, the construction of diterpenoid biosynthetic pathways in microbial cell factories is summarized. The research progress and engineering strategies for efficient microbial synthesis of diterpenoids are discussed, and the key challenges and future directions facilitating the design of high-yield diterpenoid production platforms and their translation into industrial practice are explored.

**Graphical abstract:**

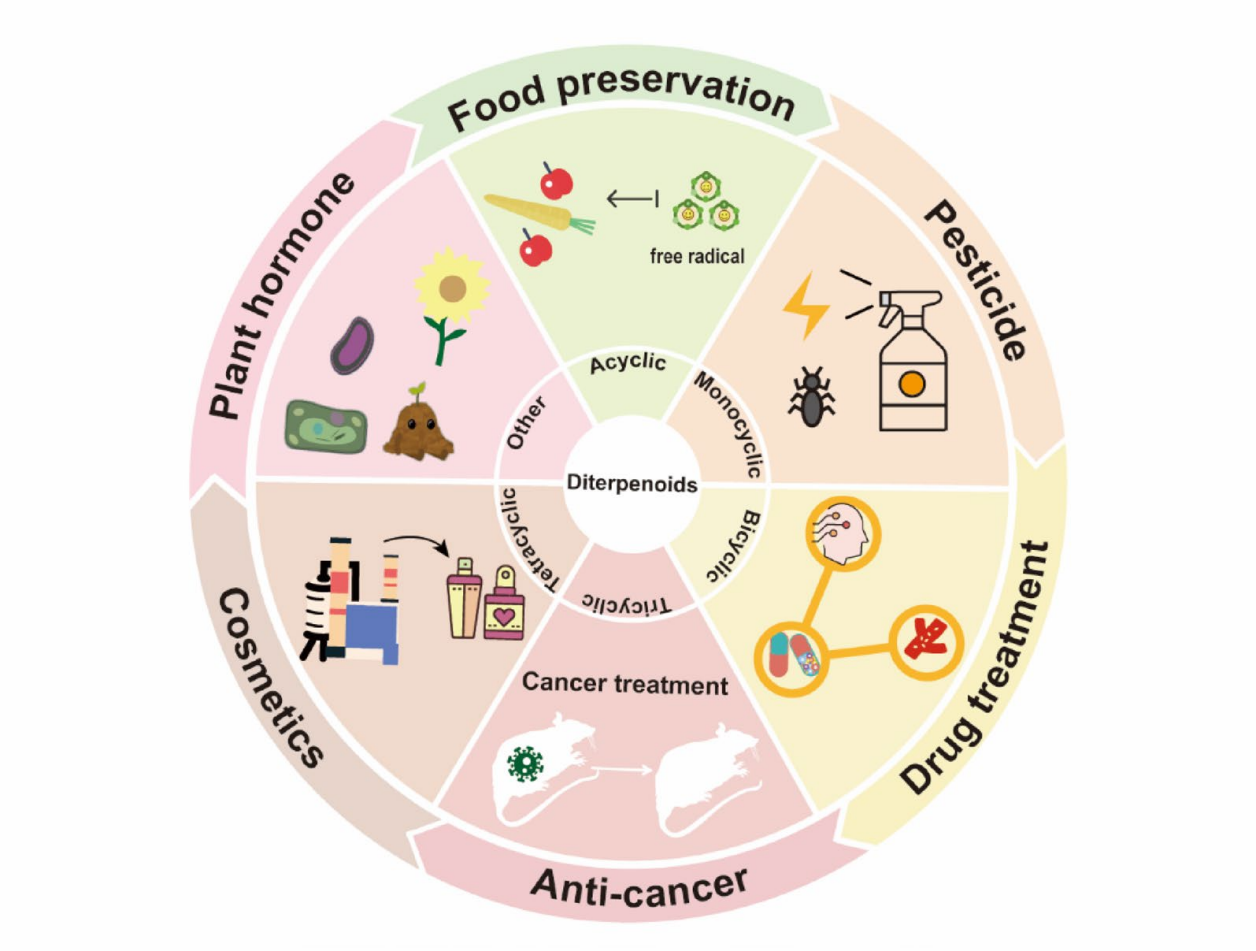

## Introduction

Diterpenoids are a class of compounds containing 20 carbon atoms, with their molecular skeletons composed of 4 isoprene units (Li et al. 2024c). Terpenoid biosynthesis originates from the two universal five-carbon precursors, isopentenyl pyrophosphate (IPP) and dimethylallyl pyrophosphate (DMAPP), which are produced via either the mevalonate (MVA) pathway in the cytosol or the methylerythritol phosphate (MEP) pathway in plastids (Ren et al. 2022; Yin and Dickschat 2023).

As plant-derived metabolites, diterpenoids are an important source of chemically and pharmacologically valuable compounds. Their complex carbon skeletons and functionalization confer a wide range of bioactivities (Nowrouzi et al. 2022; Weaver 2014). These properties enable diterpenoids to play important roles across multiple fields, including food, agriculture, and medicine. For instance, the diterpenoid phytohormone gibberellin has been efficiently produced in *Yarrowia lipolytica* to improve crop yield and quality (Kildegaard et al. 2021). Diterpenoids such as carnosic acid are also employed as natural antioxidants in meat products due to their antioxidant, antibacterial, and flavor-enhancing properties (Wei et al. 2022). Furthermore, the natural sweetener rubusoside has been proposed for commercial applications as a safe, high-intensity, low-calorie alternative to traditional sucrose and artificial sweeteners (Xu et al. 2022). Additionally, *Salvia miltiorrhiza* (Danshen) formulations containing tanshinone IIA are widely applied in the management of cardiovascular and inflammatory diseases (Zhang et al. 2012).

However, many high-value terpenoids, especially those with complex structures essential for bioactivity, are often present in low abundance in plants, making large-scale extraction difficult (Chen et al. 2021). For instance, paclitaxel accumulates at only 0.001–0.05% of the dry weight of yew bark, while the diterpenoid precursor taxadiene is not naturally accumulated in large quantities (Zhang et al. 2023). Likewise, the content of forskolin in *Coleus forskohlii* roots is relatively low and varies considerably with cultivar, growth conditions, and developmental stage (Ju et al. 2021). Conventional approaches based on plant extraction or total chemical synthesis are often unsustainable and environmentally unfriendly due to long cultivation cycles, low secondary metabolite accumulation, and high production costs, frequently resulting in limited availability of key compounds (Moser and Pichler 2019).

Microbial production is less dependent on agricultural resources and arable land, while being largely unaffected by seasonal fluctuations and environmental conditions, making it a promising route for the sustainable manufacture of diterpenoids. Advances in synthetic biology have enabled substantial progress, with a wide range of microorganisms engineered for terpenoid production (Table 1). Among them, the production titers of several high-value diterpenoids, including taxadiene, sclareol, and ent-kaurane have reached the gram-per-liter level in heterologous hosts. For instance, Hu et al. achieved the *de novo* biosynthesis of miltiradiene in yeast by increasing precursor supply and diterpenoid dehydrogenase activity (Hu et al. 2020). Beyond conventional model hosts, the non-traditional yeast *Y. lipolytica*, which has an endogenous MVA pathway and high acetyl-CoA levels, has become an attractive chassis for terpenoid biosynthesis (Zhang et al. 2023). Using this platform, Huang and co-workers established a heterologous biosynthetic pathway and integrated protein engineering with multiple metabolic engineering strategies, achieving a sclareol titer of 13.9 g/L in *Y. lipolytica*, representing the highest production level reported in this host to date (Huang et al. 2026). In addition to the widely used hosts *S. cerevisiae* and *Y. lipolytica*, several non-conventional microorganisms have also demonstrated potential for diterpenoid production. For example, the photosynthetic microalga *Chlamydomonas reinhardtii* has been engineered for the production of hydroxy-functionalized diterpenoid derivatives and heterologous diterpenoids, highlighting its potential as a sustainable platform that directly utilizes light energy and CO₂ (Lauersen et al. 2018). In addition, the oleaginous yeast *Rhodosporidium toruloides* has been successfully engineered to produce ent-Kaurene from lignocellulosic hydrolysates, demonstrating its ability to efficiently utilize renewable feedstocks while maintaining high diterpenoid production capacity (Geiselman et al. 2020). Although research on these non-conventional hosts is still less mature than that on *S. cerevisiae* and *Y. lipolytica*, their distinctive metabolic features and physiological properties make them attractive platforms for expanding microbial diterpenoid production.

**Table 1:** Representative engineering strategies for microbial production of diterpenoids

Diterpenoid	Chassis	Engineering strategy	Titer/Yield	References
Taxane diterpenoids				
Taxadiene	*Escherichia coli*	Multivariate-modular pathway engineering Module balancing through multivariate optimization	1.02 mg/L	Ajikumar et al. (2010)
Taxadiene	*Saccharomyces cerevisiae*	Fusion solubility tags Multi-copy chromosomal integration	129.00 mg/L	Nowrouzi et al. (2020)
Taxadiene	*Yarrowia lipolytica*	“Push-pull” strategy Solubilizing tags Multi-copy iterative integration	101.40 mg/L	Xu et al. (2023)
Taxadiene	*Saccharomyces cerevisiae*	Growth phase-dependent expression control Subcellular protein localization Altering protein turnover or solubility	20.00 mg/L	Reider Apel et al. 2017)
Oxygenated taxanes	*Saccharomyces cerevisiae*	Upstream pathway reconstruction and optimization Optimization of the high-throughput microscale PH control	78.00 mg/L	Walls et al. (2020)
Oxygenated taxanes	*Saccharomyces cerevisiae*	Uncoupling reaction Testing five cofactors involved in the structural components of their enzymes Resting cell assays	361.40 ± 52.40 mg/L	Nowrouzi et al. (2022)
Kaurane-derived diterpenoids				
ent-Kaurene	*Escherichia coli*	GGPPS and host strain screening Constructed and expressed CPS and KS Culturing strain in a bioreactor	578.00 mg/L	Kong et al. (2015)
Gibberellins	*Yarrowia lipolytica*	Expression the heterologous biosynthetic enzymes Protein engineering Expression of additional ent-Kaurenoic acid biosynthetic genes	12.81 mg/L	Kildegaard et al. (2021)
Steviol	*Escherichia coli*	Modification of transmembrane CYP oxidases Co-expression of cytochrome b5 AlphaFold-based protein engineering	1.07 g/L	Sun et al. (2022)
Rubusoside	*Saccharomyces cerevisiae*	Inserting the KS Overexpression of *tHMG1* and *IDI1* Overexpression of mutant *FPS*^F112A^ Truncating the transmembrane domain of CPR Efflux system engineering Stress-responding regulator	1.36 g/L	Xu et al. (2022)
Labdane diterpenoids				
Sclareol	*Saccharomyces cerevisiae*	Engineering sclareol synthase Metabolic rewiring for sclareol biosynthesis Transcriptional analysis Fed-batch fermentation	11.40 g/L	Cao et al. (2023)
Sclareol	*Yarrowia lipolytica*	Engineering heterologous Lpps and Scs GGPP pathway enhancement and enzyme colocalization Construction scaffold-free multienzyme complexes	12.90 g/L	Sun et al. (2024)
Sclareol	*Chlamydomonas reinhardtii*	Screening for potential GGPPs Enzymatic fusion of GGPPS to diTPS Overexpression of the main rate-limiting enzyme DXR in the MEP pathway Photoautotrophic cultivation strategies	656. 00 mg/L	Einhaus et al. (2022)
Sclareol	*Yarrowia lipolytica*	Semirational mutagenesi Pathway balancing Transporter engineering Central carbon metabolism engineering	13.90 g/L	Huang et al. (2026)
Sclareol	*Yarrowia lipolytica*	Fusion protein Expressing of heterologous GGPP synthase Promoter engineering Overexpression of key enzymes in the MVA pathway Acetyl-CoA supply enhancement and gene dosage optimization	2656.20 mg/L	Chen et al. (2025)
Sclareol	*Pichia pastoris*	Enzyme fusion engineering Pathway optimization Global transcriptional rewiring Carbon and nitrogen flux balancing Cell robustness engineering	27.80 g/L	Zhang et al. (2026)
(13R)-Manoyl oxide	*Saccharomyces cerevisiae*	Expressing t*Cf*TPS2 and t*Cf*TPS3 Fusion protein Overexpression of TPS	3.00 g/L	Zhang et al. (2019)
(13R)-Manoyl oxide	*Chlamydomonas* *reinhardtii*	Expression of heterologous plant diTPS Codon optimization of TPS and CYP Intron-contain transgenes Truncating CYPs	80.00 mg/g	Lauersen et al. (2018)
Forskolin	*Saccharomyces cerevisiae*	Fusion protein Overexpression of *HMG1* Truncating the N terminus of *Cf*CPR ER and cofactor engineering Fed-batch fermentation	21.47 mg/L	Ju et al. (2022)
Abietane diterpenoids				
Miltiradiene	*Saccharomyces cerevisiae*	Overexpression of the pathway genes Transcription factor engineering Protein modification of *Sm*KSL1	3.50 g/L	Hu et al. (2020)
Ferruginol	*Corynebacterium glutamicum*	Targeting metabolic pathway modifications Overexpression the key enzymes of MEP pathway Modular gene expression of heterologous metabolic pathways	107.34 mg/L	Lee et al. (2025)
Carnosic acid	*Saccharomyces cerevisiae*	Overexpression of *BTS1*-GGGS-*ERG20*^F96C^ Integrating the genes encoding CYPs and CPR *Sm*CPR Coexpression of fusion proteins ER and cofactor optimization	75.18 mg/L	Wei et al. (2022)
Levopimaradiene	*Escherichia coli*	Improvement of metabolic precursor supply Protein engineering Site-directed mutation of TPS and GGPPS	700.00 mg/L	Leonard et al. (2010)
Levopimaric acid	*Saccharomyces cerevisiae*	Overxpression of *tHMG1*, *IDI1*, *BTS1*-GGGS-*ERG20*p N-terminal truncating and site-directed mutagenesis Co-expression of three CPRs	400.31 mg/L	Liu et al. (2018a)
Cembrane diterpenoids				
Cembratriene-ol	*Saccharomyces cerevisiae*	Heterologous pathway reconstruction Expression of the *ERG20* Dynamic control of the *ERG9* Mitochondrial compartmentalized expression	284.00 ug/L	Yang et al. (2023a, b)
Cembratriene-ol	*Escherichia coli*	Expressing of heterologous *CrtE* and *CBTS* Overexpression of cbts*, *dxs*, *idi*, *ispA*, *ispD*, and *ispF*	371.20 mg/L	Yang et al. (2023a, b)
Clerodane diterpenoids				
ent-Copalol	*Saccharomyces cerevisiae*	Strengthening the mevalonate pathway genes Weakening the competing pathway Enzyme mining via transcriptomics Precursor enhancement and enzyme fusion Protein engineering	35.60 mg/L	Li et al. (2024d)
Andrographolide	*Andrographis Paniculata*	Methyl jasmonate (MeJA) treatment Mining cytochrome CYPs Co-expression of CYP and *Ap*CPS2 Screening the key enzymes for the biosynthesis of andrographolide Combined expression of different enzymes		Wang et al. (2025)
Other diterpenoids				
Geranylgeraniol	*Saccharomyces cerevisiae*	Overexpression of the *HMG1*, *BTS1*-*DPP1* and *BTS1*-*ERG20* Multicopy integration vectors	3.31 g/L	Tokuhiro et al. (2009)
Geranylgeraniol	*Yarrowia lipolytica*	Expressing heterologous phosphatase genes Enhancing precursor supplies Promoter engineering Reducing reactive oxygen species	3.35 g/L	Wang et al. (2024)
cis-Abienol	*Escherichia coli*	Coexpression of the MVA and MEP pathways Screening for highly efficient cis-Abienol synthase Two-phase cultivation	634.70 mg/L	Li et al. (2019)
cis-Abienol	*Escherichia coli*	Screening and coordinating of alcohol kinases and IPKs Constructing a double-phosphatase-deficient strain Changing the IOH/DMAOH addition strategy Optimization of the fed-batch fermentation	1.38 g/L	Zhang et al. (2022)
Lathyranes	*Saccharomyces cerevisiae*	Optimizing the expression of CYPs and alcohol ADH Protein tagging strategies Coexpressing of ElC9OX1p and JcC5OX2p alongside an ADH Codon-optimization of CYPs	800.00 mg/L	Wong et al. (2018)

CPS: ent-Copalyl diphosphate synthase; KS: ent-Kaurene synthase; GGPPS: geranylgeranyl diphosphate synthase; CYP: cytochrome P450 monooxygenase; FPS: farnesyl diphosphate synthase; CPR: cytochrome P450 reductase; Lpps: labdenediol diphosphate synthase; Scs: sclareol synthase; diTPS: diterpenoid synthase; ER: endoplasmic reticulum; IPK: isopentenyl phosphate kinase; IOH: isoprenol; DMAOH: dimethylallyl alcohol; ADH: alcohol dehydrogenase

Although considerable advances have been made in microbial diterpenoid production, the development of efficient production platforms is further complicated by lengthy and highly branched pathways, demanding enzyme cascades, challenges associated with process scale-up, and the cost of downstream product recovery. In recent years, research efforts have increasingly shifted beyond simple pathway assembly toward more sophisticated engineering strategies.

While previous reviews have primarily summarized diterpenoid diversity, biosynthetic mechanisms, enzyme discovery, and representative production examples, a comprehensive evaluation of the engineering challenges and solutions associated with microbial diterpenoid production is still lacking. Recent advances in synthetic biology have provided a growing toolbox for overcoming limitations in enzyme activity, precursor availability, pathway efficiency, and product accumulation. Accordingly, this review focuses on the engineering strategies that underpin successful diterpenoid biomanufacturing, including enzyme optimization, precursor supply engineering, compartmentalized biosynthesis, dynamic pathway regulation, and host engineering. Rather than cataloging diterpenoid products, we emphasize the design principles and technological approaches that have enabled substantial improvements in production performance. These advances highlight the potential of microbial cell factories as sustainable platforms for the large-scale manufacture of high-value diterpenoids.

## Biosynthetic pathways of diterpenoids

**Fig. 1:**
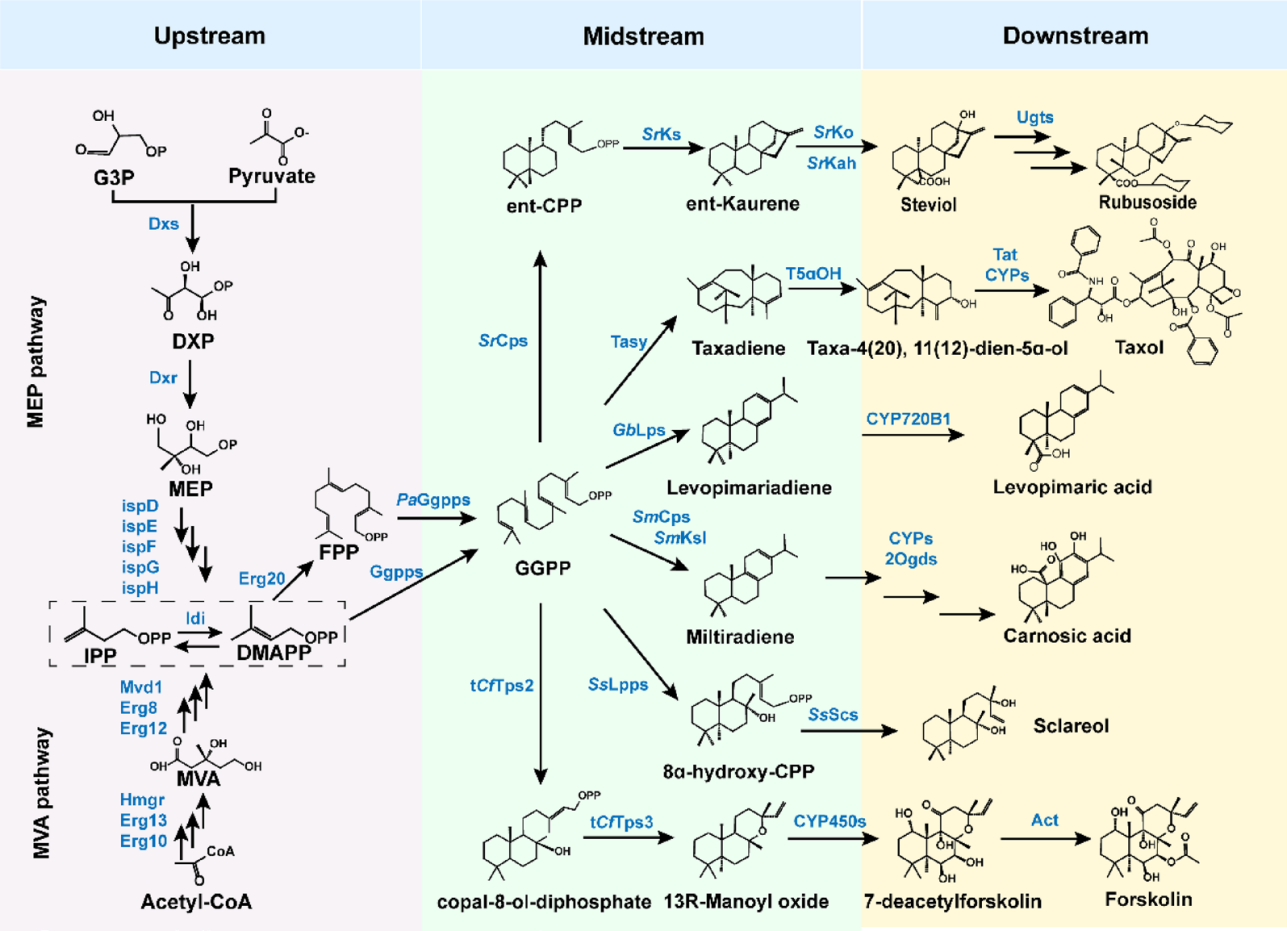
Biosynthetic pathways of diterpenoids in microbes. Ent-CPP: ent-copalyl diphosphate; *Sr*Cps: *Stevia rebaudiana* copalyl diphosphate synthase; *Sr*Ks: *Stevia rebaudiana* kaurene synthase; *Sr*Ko: *Stevia rebaudiana* ent-Kaurene oxidase; *Sr*Kah: *Stevia rebaudiana* kaurenoic acid 13α-hydroxylase; Ugts: Uridine diphosphate glycosyltransferase; T5αOH: Taxadien-5α-ol hydroxylase; Tat: Taxadien-5α-ol O-acetyltransferase; *Gb*Lps: *Ginkgo biloba* levopimaradiene diterpene synthase; *Ss*Lpps: *Salvia sclarea* labdenediol diphosphate synthase; *Ss*Scs: *Salvia sclarea* sclareol synthase; *Sm*Cps: *Salvia miltiorrhiza* copalyl diphosphate synthases; *Sm*Ksl: *Salvia miltiorrhiza* kaurene synthase like; *Cf*Tps2: *Coleus forskohlii* diterpenoid synthase 2; *Cf*Tps3: *Coleus forskohlii* diterpenoid synthase 3

The core skeleton of diterpenoid compounds consists of four isoprene units (C5), and fulfills diverse biological functions, including roles in cell integrity, hormone signaling, electron transport, and photosynthesis (Gao et al. 2021). In plants, diterpenoid compounds are biosynthesized via the MVA and MEP pathways, where IPP and DMAPP condense to form the direct diterpenoid precursor geranylgeranyl diphosphate (GGPP). Subsequently, diterpenoid synthases catalyze the cyclization reaction of GGPP to form diverse hydrocarbon skeletons. The structural diversity of these skeletons directly influence biological function. Finally, these hydrocarbon scaffolds are further diversified through a series of tailoring reactions catalyzed by cytochrome P450 monooxygenases (CYPs), flavin-dependent monooxygenases, UDP-glycosyltransferases (UGTs) and acyltransferases, leading to the formation of structurally diverse diterpenoid molecules (Fig. 1) (Dinday and Ghosh 2023; Lipko et al. 2023).

### Scaffolds formation of diterpenoid by diterpenoid synthases

Diterpenoid synthases (diTPS) catalyze the conversion of GGPP into structurally diverse diterpenoid scaffolds, representing one of the most challenging chemical steps in the biosynthesis of diterpenoid products. The classical classification system divides these enzymes into two types: class I enzymes, which cleave the pyrophosphate ester bond through the DDXXD motif, and class II enzymes, which initiate protonation-induced cyclization via the DXDD motif (Pan et al. 2024; Wendt and Schulz 1998). Despite differences in their catalytic initiation mechanisms, both enzyme classes achieve product formation through the precise control of reactive carbocation intermediates. As structural and mechanistic studies continue to uncover the molecular basis of terpene cyclization, attention has increasingly shifted from enzyme classification toward understanding the determinants of product specificity and exploiting this knowledge for enzyme engineering and pathway optimization.

The catalytic selectivity of diTPS is influenced not only by the amino acid composition of the active site but also by the geometric configuration of the substrate(Yin et al. 2026). Experimental approaches alone often provide limited information on the transient carbocation intermediates that govern diterpene cyclization reactions (Tang et al. 2017). To overcome this challenge, computational methods have become increasingly important for probing reaction pathways at the atomic level. In particular, the combination of quantum me3chanics/molecular mechanics (QM/MM) simulations, isotope-labeling experiments and density functional theory (DFT) calculations has greatly advanced mechanistic studies of diTPS (Xu et al. 2021). An elegant example was recently reported by the Dickschat group. By changing the C6 double bond of GGPP from the natural E-configuration to the Z-isomer, the authors observed striking shifts in the product spectra of multiple diTPSs and generated a series of previously undescribed carbon skeletons. Mechanistic analysis further revealed that the altered substrate geometry reshaped the carbocation reaction network, ultimately redirecting cyclization trajectories and product formation (Yin et al. 2026). This work underscores the importance of substrate stereochemistry as a determinant of diTPS product specificity and provides a new framework for generating diterpenoid structural diversity through substrate engineering.

- The combination of DFT calculations and chemical transformations has also revealed the underlying logic of complex scaffold formation. Chen et al. conducted a computationally guided exploration of the cis- and trans-eunicellane scaffolds formed by two bacterial diTPSs, Bnd4 and AlbS. DFT calculations indicated that, despite only differing in the configuration at C1, the chemical and thermodynamic properties of the two scaffolds are markedly different. Leveraging the reactivity of the trans-eunicellane scaffold, the study further synthesized a series of gersemiane-type diterpenoids through electrophilic cyclization, achieving a complete closed loop from computational prediction to scaffold derivation (Li et al. 2024d).
- A deeper understanding of the regulatory mechanisms involved is being systematically applied to enzyme engineering for the functional reprogramming of diterpenoid scaffolds. The phenomenon of altering product profiles through single-point mutations has been confirmed in multiple diterpenoid synthase systems. Peters and colleagues identified two diterpenoid synthases, *Ir*KSL3a and *Ir*TPS2, from *Isodon rubescens*, which share 98% sequence homology but employ distinctly different carbocation quenching strategies. Through systematic site-directed mutagenesis and multi-scale QM/MM simulations, the researchers successfully engineered *Ir*KSL3a to produce nezukol and other hydroxylated products (Jin et al. 2024). This work identified key molecular determinants governing the transition of diTPS activity from olefin production to hydroxylated product formation, providing a useful basis for future enzyme engineering efforts.

### Diterpenoid diversification mediated by CYPs oxidation and GT-mediated glycosylation

DiTPSs establish the core carbon frameworks of diterpenoids, whereas the remarkable structural diversity observed in nature is largely generated through downstream tailoring reactions. Following scaffold formation, diterpenoids are frequently subjected to oxidation, hydroxylation, epoxidation, and glycosylation, yielding compounds with distinct physicochemical and biological properties. CYPs and UGTs are among the most important enzyme families responsible for these modifications. By introducing oxygenated functional groups and sugar moieties, respectively, they reshape diterpenoid structures and contribute substantially to the diversity of naturally occurring diterpenoid metabolites (Zhao et al. 2025).

CYPs play a central role in the diversification of diterpenoid structures by catalyzing a broad spectrum of oxidative reactions. Through the introduction of oxygen-containing functionalities, these heme-dependent enzymes create new chemical handles that enable further downstream modifications. In plants, diterpenoid-related CYPs are predominantly distributed among the CYP71, CYP85, and CYP72 families, reflecting their evolutionary expansion alongside the diversification of diterpenoid metabolism (Bathe and Tissier 2019). Increasing attention is now being directed toward the coordinated use of multiple CYPs to construct tailored oxidation pathways, offering new opportunities for the biosynthesis of structurally complex and previously inaccessible diterpenoids.

A representative example comes from the abietane diterpenoid pathways found in Salvia and Rosmarinus species. By reconstructing the corresponding CYP oxidation network in yeast, Bathe et al. demonstrated that members of the CYP76AH and CYP76AK families could introduce oxidative modifications at different positions of the common intermediate abietatriene. Combinatorial expression of these enzymes generated a matrix of oxidation patterns and led to the production of 14 diterpenoid metabolites, including eight compounds that had not been previously reported (Bathe et al. 2019). This expansion of the CYP substrate scope and the engineering of CYPs not only reveal the core drivers of oxidative diversity in diterpenoids but also open up viable pathways for the discovery of novel diterpenoid scaffolds.

UGTs constitute a prominent group of enzymes responsible for the glycosylation of diterpenoid metabolites. These enzymes catalyze the transfer of sugar moieties from activated UDP-sugar donors to specific acceptor molecules, resulting in the formation of glycosylated products with enhanced physicochemical characteristics, such as increased aqueous solubility, improved stability and greater bioavailability. In many cases, glycosylation also contributes directly to the biological activity of diterpenoid natural products (He et al. 2022; Liu et al. 2025). Insights into the molecular basis of diterpenoid glycosylation were greatly advanced by the structural characterization of the steviol glycosyltransferase *Sr*UGT76G1 (Liu et al. 2020). Structural and mutational analyses revealed that even a single amino acid substitution can substantially alter enzyme selectivity and catalytic outcome. These studies demonstrated that substrate recognition is governed by subtle differences in the architecture of the active site, where pocket geometry and steric constraints influence substrate positioning and ultimately determine product regioselectivity. In 2024, it was found that the V155T substitution mutation in UGT91D2 further improved the glycosylation efficiency of steviol glycosides Reb D and Reb M. This residue, which is located near the binding sites of the sugar donor and sugar acceptor, enhances the overall net yield of the synthetic pathway by optimizing UDP-glucose binding and reducing side reactions caused by non-glucose donors (Shoji et al. 2025).

The growing availability of structural and multi-omics datasets has greatly expanded our understanding of CYP- and UGT-mediated diterpenoid diversification, enabling more informed approaches to enzyme discovery and engineering.

## Screening and engineering of diterpenoid synthases

**Fig. 2:**
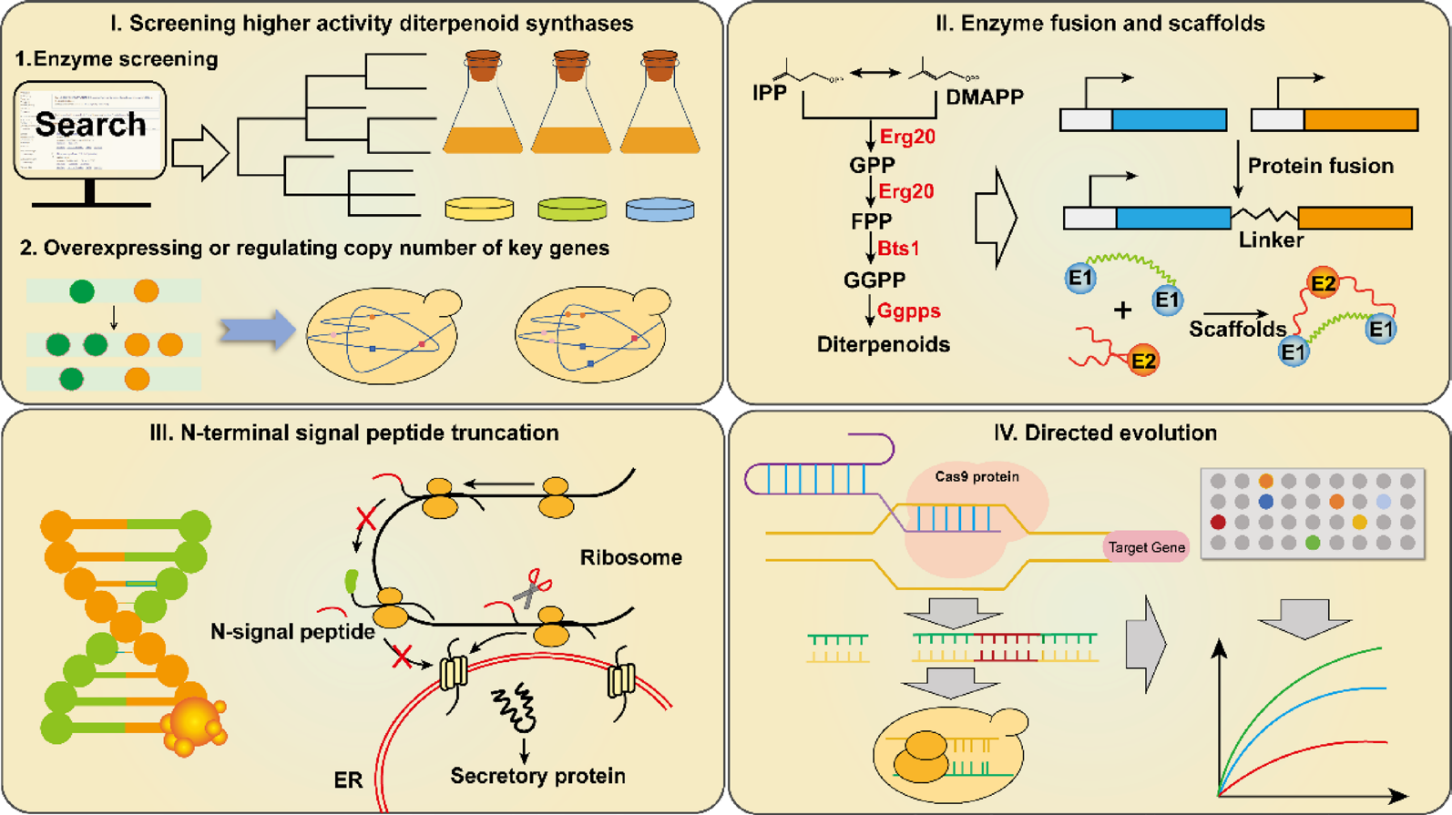
Screening and engineering of diterpenoid synthases. (I) Screening for highly efficient diterpenoid synthases. (II) Protein fusion of key enzymes of the pathway. (III) Truncating the transmembrane domain at the N-terminal of the key enzyme. (IV) Directing evolution strategy

The establishment of a functional diterpenoid biosynthetic pathway in a microbial host depends largely on the successful implementation of diTPSs responsible for scaffold formation. These enzymes catalyze complex carbocation-mediated cyclization reactions that generate the structural framework of diterpenoid products (Tholl 2015). Identifying and acquiring effective exogenous genes is critical for establishing functional metabolic pathways (Peralta-Yahya et al. 2011). Challenges such as low catalytic efficiency, poor expression, precursor limitation, and product toxicity frequently constrain pathway output. As a result, considerable effort has been devoted to the identification of more effective enzymes and the development of protein engineering approaches to enhance their performance in microbial production systems (Fig. 2).

### Screening of highly efficient terpenoid synthase

Plant diTPSs catalyze the cyclization of GGPP into various cyclic skeletons. Their cyclization mode directly determines the structural and stereochemical diversity of diterpenoids (Jia et al. 2017). Therefore, mining novel terpenoid synthases from diverse sources enriches structural diversity and aids in discovering new bioactive compounds (Bian et al. 2017). Furthermore, the investigation into the substrate preference mechanisms of diTPS is essential. Structural features of the active site, along with the regulatory roles of key amino acid residues, critically determine the enzyme’s substrate recognition specificity and catalytic efficiency (Li et al. 2023).

For organisms with sequenced genomes, candidate genes can be identified via homology-based screening using public databases (e.g., NCBI, JGI) or proprietary data, often by BLAST analysis against conserved motifs (e.g., DDXXD/E, NSE/DTE). Nonetheless, a substantial proportion of biosynthetically promising diterpene synthases in nature remain uncharacterized, residing within genomes that are either unsequenced or functionally unannotated.

Systematic mining of novel diTPSs from diverse plants is a promising strategy to expand the chemical space. For example, in a study targeting the biosynthesis of forskolin, Pateraki et al. used transcriptomic data from *C. forskohlii* root cultures to identify candidate diTPS genes *Cf*TPS2 and *Cf*TPS3 (Pateraki et al. 2014). Furthermore, RNA sequencing was subsequently employed to screen and characterize the CYPs involved in the later modifications of forskolin. Similarly, Hu et al. screened diTPSs from various species and found that a chimeric synthase combining class II *Cf*TPS1 (*Scutellaria pilosula*) and class I *Sm*KSL1 (*Salvia miltiorrhiza*) exhibited the highest efficiency for diterpenoid production in yeast (Hu et al. 2020). Kirby et al. explored diTPSs from Euphorbiaceae plants for heterologous production of neocembrene and casbene in yeast (Kirby et al. 2010). Sun et al. screened lysophosphatidic acid phosphatases (*Cc*Cls, *Ss*Lpps) from different plants and co-expressed them with sclareol synthase in *Y. lipolytica* for *de novo* sclareol production (Sun et al. 2024). Zerbe et al. identified five candidate diTPSs in *Marrubium vulgare* via metabolite and transcriptome analysis; co-expression of *Mv*CPS1 and *Mv*ELS in engineered *E. coli* and *Nicotiana benthamiana* enabled production of precursors for bioactive diterpenoids (Zerbe et al. 2014).

Recent advances in artificial intelligence have provided new opportunities for diTPS discovery and characterization. By integrating large-scale sequence datasets with machine learning algorithms, AI-based approaches can facilitate enzyme identification, functional annotation, and protein engineering, thereby accelerating the exploration of diterpenoid biosynthetic diversity (O’Leary 2024).

### Enzyme fusions and scaffolds

The activity of heterologous terpenoid synthases varies across microbial hosts due to structural or catalytic differences, directly affecting production titers. Fusion protein engineering facilitates substrate channeling by bringing sequential catalytic modules into close proximity, thereby enhancing intermediate transfer efficiency and promoting metabolic flux toward the desired product (Liu et al. 2023b).

The longer the reconstructed pathway, the greater the metabolic burden on cells, and the higher the likelihood of unintended crosstalk between synthetic and native metabolic pathways, pathway dysregulation, or even toxicity. To address the limitations of terpenoid production in yeast, strategies have been developed to enhance enzyme proximity and substrate availability, thereby improving catalytic efficiency. The simplest strategy in research to increase enzyme proximity and promote substrate channeling is enzyme fusion (Fig. 2) (Ignea et al. 2015a, b). Zhou et al. used pathway modularization in *S. cerevisiae* and fused miltiradiene synthases (*Sm*CPS1 and *Sm*KSL1), achieving a titer of 365.0 mg/L in a 15-L fermenter (Zhou et al. 2012). The synthesis of GGPP in microorganisms involves two key enzymes farnesyl diphosphate synthase (FPPS) and geranylgeranyl diphosphate synthase (GGPPS), which may become a limiting factor for diterpenoid yields. To overcome the potential limitations of modular GGPP biosynthesis in yeast, Codruta Ignea et al. constructed a fusion between ERG20p and GGPPS (Ignea et al. 2015a, b). The expression of the reverse fusion protein increased the production of GGPP by 7 times, reaching 6.8 mg/L. Cao et al. enhanced sclareol production by spatially coupling the class I and II diterpenoid synthases (Tps and Lpps) from *Salvia sclarea*, resulting in a 6.7-fold increase in sclareol titer. To further improve protein performance, a maltose-binding protein (MBP) was fused to the N-terminus of the Tps-Lpps construct (MBP-TL), which increased enzyme stability and contributed to more efficient biosynthesis (Cao et al. 2023). Additionally, Xu et al. demonstrated that fusing a SUMO solubility-enhancing tag to the N-terminus of Tasy significantly increased the titer of taxadiene (Xu et al. 2023). In addition, most plant-derived cytochrome CYPs exhibit low expression efficiency in microbial hosts. Engineering CYP-CPR fusion proteins can enhance electron transfer efficiency, improving yields of high-value compounds such as paclitaxel and forskolin (Ajikumar et al. 2010; Ju et al. 2021). Enzyme linkage and subsequent co-localization enhance substrate channeling through the pathway. The proximity of substrate-binding sites to the catalytic sites of upstream enzymes reduces competition for common precursors by endogenous enzymes and minimizes interference with required reactions (Kunhao et al. 2026).

Although conceptually simple, enzyme fusion applications are complex. Improper linker design can cause misfolding or loss of function, requiring optimization of linker length and composition (Chen et al. 2013). For example, in the biosynthesis of forskolin, th ree distinct linkers were tested for fusing *Cf*CYP76AH15 and *Cf*CPR. The results indicated that only the fusion protein containing the “GGG” linker led to an increased yield of forskolin (Ju et al. 2022). In recent work, Sun et al. used a pair of short peptides (RIDD/RIAD) to construct multi-enzyme complexes, alleviating metabolic imbalance in the production of sclareol with a 2:1 stoichiometry (Sun et al. 2024).

### N-terminal signal peptide truncation

While fusion protein engineering can enhance pathway performance by improving substrate channeling and coordinating sequential catalytic reactions, the efficient heterologous expression of plant-derived diTPSs remains another important challenge. Many diTPSs contain N-terminal plastid-targeting peptides that mediate protein transport into plastids in their native plant hosts. During this process, the transit peptides are typically removed by plastid-localized processing peptidases. However, such processing machinery is absent in microbial hosts such as yeast. Consequently, the retention of these nonfunctional peptide sequences may impair protein folding and reduce catalytic efficiency. Although artificial truncation of signal peptides predicted by bioinformatics tools is a feasible strategy to enhance the expression activity of diTPSs, the truncation length remains a critical factor influencing enzyme catalytic activity.

3-Hydroxy-3-methylglutaryl-CoA reductase (Hmgr) catalyzes an irreversible step in the MVA pathway and serves as a major control point for carbon flux toward terpenoid biosynthesis (Yang et al. 2016; Zhou et al. 2012). The N-terminus of Hmgr contains a domain that mediates protein degradation. Previous studies have demonstrated that truncating the N-terminal transmembrane domain of HMG-CoA reductase (resulting in tHMGR) while retaining its C-terminal catalytic domain can prevent N-terminal transmembrane domain-mediated self-degradation and increase its solubility in the cytoplasm (Fig. 2) (Donald et al. 1997). Wei et al. truncated the N-terminal of *Sm*CPS1 and *Sm*KSL1 genes and constructed fusion proteins. The results showed that when the N-terminal truncated t*Sm*CPS1 and t*Sm*KSL1 were fused with the linker peptide GSTSSGSSG, the yield of tanshinone dienone was the highest (Wei et al. 2022).

Most plant-derived CYPs and their associated CPRs are naturally anchored to cellular membranes through N-terminal hydrophobic segments. Typically, modification or truncation of these membrane-targeting sequences is frequently employed to improve protein expression and catalytic performance (Lauersen et al. 2018; Zhao et al. 2016). Using the TMHMM software for prediction, Ju et al. identified an N-terminal transmembrane region comprising 66 amino acids in *Cf*CPR. Subsequent truncation of this region demonstrated that removal of the transmembrane domain facilitates electron transfer in *Cf*CPR (Ju et al. 2022). These results indicate that truncating N-terminal domain of certain enzymes can effectively increase the yield of the products.

### Directed evolution

TPSs are frequently targeted in the heterologous anabolic regulation of terpenoids due to their remarkable catalytic versatility, enabling the formation of thousands of distinct molecules. However, many naturally occurring enzymes perform poorly in heterologous hosts, limiting pathway productivity. Although enzyme discovery and expression optimization can improve production to some extent, further enhancement often requires protein engineering. Therefore, advanced protein engineering strategies, including directed evolution, rational design, and semi-rational design are essential to enhance enzyme performance (Furubayashi et al. 2014).

Directed evolution has emerged as a versatile tool for protein optimization, relying on the creation of diverse mutant libraries followed by the identification of variants exhibiting enhanced catalytic or physicochemical properties (Arnold 2018). The overarching objective is to emulate the natural evolutionary process of enzymes under controlled laboratory conditions. This involves conducting multiple cycles of targeted gene mutation, expression, and screening. The aim is to accelerate the evolutionary timeline, condensing millennia of natural evolution into a significantly shorter period. Ultimately, this approach seeks to yield enzymes with novel functionalities and enhanced performance (Sheldon and Pereira 2017).

To enhance the performance of TPSs beyond their native capabilities, directed evolution strategies such as error-prone PCR (ep-PCR), iterative saturation mutagenesis (ISM), and staggered extension process (StEP) have been widely adopted. These approaches generate diverse enzyme repertoires that can be screened for desirable traits, thereby accelerating the identification of functionally important regions and beneficial mutations. By modifying key parameters of the native enzyme, these approaches enhance catalytic performance, ultimately enabling efficient and targeted heterologous terpenoid synthesis in microbial cells (Fig. 2) (Leonard et al. 2010). Among these techniques, ep-PCR represents a convenient and widely adopted strategy for introducing random mutations across an entire gene sequence. The mutation frequency can be tuned through modifications of PCR conditions, including altered Mg²⁺ levels and the use of error-prone polymerases. For example, Lauchli et al. generated a library of roughly 2,800 BcBOT2 variants and successfully isolated a thermostable TPS that maintained its native catalytic function, demonstrating the utility of ep-PCR for improving enzyme robustness without compromising activity (Lauchli et al. 2013).

In contrast, rational or semi-rational design guides the selection of mutation sites based on partial structural information. Rational design strategies are commonly employed to alter enzyme substrate or product specificity, while semi-rational approaches are typically applied to optimize the active site or substrate-binding pocket (Liang et al. 2025). To improve the activity of levopimaradiene synthase (Lps), researchers adopted a semi-rational design strategy that combined evolutionary information with active-site analysis. Amino acid residues predicted to influence substrate interactions were individually modified, and the resulting variants were characterized in *E. coli*. Several mutations were found to positively affect enzyme efficiency, demonstrating the effectiveness of this targeted engineering approach (Liu et al. 2018a).

Despite its effectiveness, random mutagenesis-based directed evolution often generates extensive variant libraries in which beneficial mutations represent only a small fraction of the population. Consequently, in the absence of robust high-throughput screening or selection systems, identifying improved mutants from a large genetic background can become labor-intensive and time-consuming. In addition, screening is also the bottleneck that restricts the directed evolutionary transformation of enzymes. How to efficiently modify the directed evolutionary strategies of multiple enzymes, construct high-quality diverse mutation libraries and efficient and rapid screening methods will be the direction of future efforts.

## Engineering strategies for efficient microbial synthesis of diterpenoids

### Enhancing the MVA or MEP pathway

The MVA pathway is subject to complex transcriptional and post-transcriptional regulation, which often constrains the metabolic flux toward terpenoid precursor biosynthesis. To overcome this bottleneck, metabolic engineering strategies have predominantly focused on overexpressing key genes within the MVA pathway to enhance precursor availability and drive the accumulation of target terpenoids.

Traditionally, most metabolic engineering efforts have utilized well-established model organisms due to their well-characterized genetics and predictable metabolic behavior. However, non-conventional strain *Y. lipolytica* have recently emerged as promising platforms for isoprenoid biomanufacturing. The advantages of *Y. lipolytica* include high endogenous acetyl-CoA levels, robust cofactor synthesis, and the presence of a native MVA pathway, which makes it an attractive alternative for large-scale terpenoid production.

**Fig. 3:**
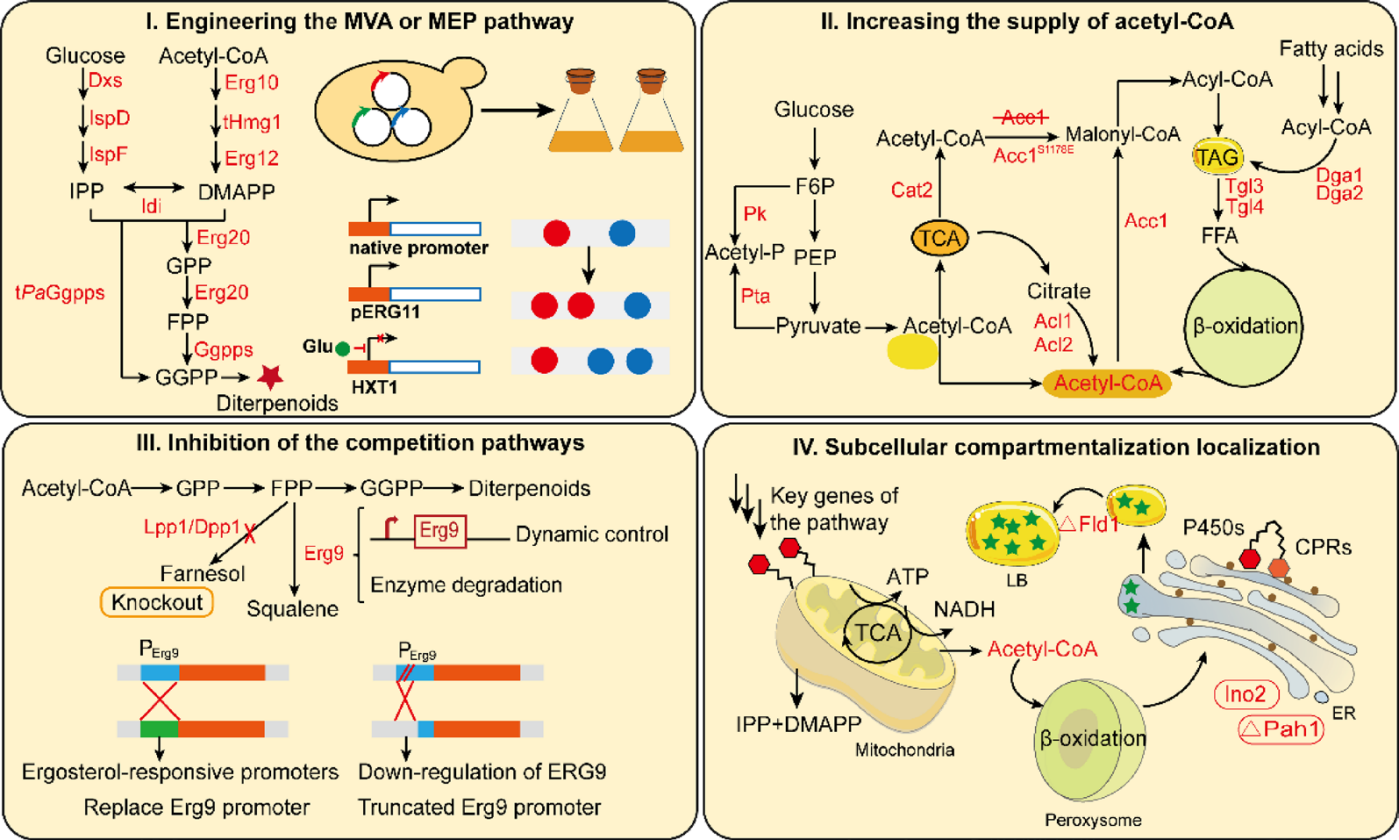
Engineering strategies for efficient synthesis of diterpenoids. (I) Engineering the MVA or MEP pathway. (II) Increasing the supply of acetyl-CoA. Enhancing cytosolic acetyl-CoA flux and the utilization of acetyl-CoA from fatty acid β-oxidation. (III) Inhibition of competing pathways. Downregulating squalene synthesis by replacing with responsive promoter or truncating promoter. (IV) Subcellular compartmentalization

In the MVA pathway, acetyl-CoA is sequentially converted into FPP through eight enzymatic steps catalyzed by *ERG10*, *ERG13*, *HMG1*, *ERG12*, *ERG8*, *ERG19*, *IDI*, and *ERG20*. Previous studies have demonstrated that overexpressing these genes can enhance flux through the MVA pathway, thereby boosting terpenoid production (Fig. 3) (Gao et al. 2017). Given its position at a key regulatory step of the MVA pathway, HMGR is frequently targeted in metabolic engineering strategies to enhance carbon flux toward terpenoid synthesis (Tokuhiro et al. 2009). Xu et al. demonstrated that overexpressing both t*HMG1* and *GGSP1* in *S. cerevisiae* led to a 286% increase in the titer of taxadiene (Xu et al. 2023). A common strategy for improving diterpenoid production in *S. cerevisiae* is to increase the intracellular pool of GGPP while simultaneously reducing carbon loss to sterol biosynthesis. Downregulation of *ERG9*, achieved by replacing its native promoter with the weaker *ERG11* promoter, decreases flux toward squalene formation and preserves FPP for terpenoid biosynthesis (Wang et al. 2024). In addition, Bts1p catalyzes the condensation of FPP and isopentenyl diphosphate (IPP) to generate GGPP. Consequently, overexpression of *BTS1* is frequently employed to enhance GGPP supply and improve diterpenoid titers (Ignea et al. 2015a, b). Similarly, Chen et al. increased the yields of GGPP and sclareol by integrating the key enzymes in *Y. lipolytica* (Chen et al. 2025).

Increased production of terpenoid compounds in engineered microorganisms can be achieved by employing a “push-pull” strategy (Lee et al. 2025). In *cyanobacteria*, terpenoid biosynthesis relies solely on the MEP pathway for precursor generation. To increase the availability of GGPP, additional copies of *Cf*DXS and *Cf*GGPPS were integrated into the chromosome and co-expressed. This metabolic engineering strategy enabled the recombinant strain to accumulate up to 0.45 mg g⁻¹ DCW of (13R)-Manoyl oxide (Englund et al. 2015). Ajikumar et al. used multi-module metabolic engineering to build a factory of taxidiene-producing cells in *E. coli*, overexpressing four key enzymes in the MEP pathway, Dxs, Idi, IspD and IspF, and enhancing downstream pathway genes on the basis of efficient GGPP productionv (Ajikumar et al. 2010). The titer of taxol precursor taxadiene is 1.0 g/L. Given that the MEP pathway inherently channels a high carbon flux toward photosynthetically active pigments, redirecting metabolic flux through the MEP pathway in *C. reinhardtii* represents an effective strategy for enhancing diterpenoid production (Einhaus et al. 2022).

Achieving higher diterpenoid titers may depend on the integrated optimization of both the MVA and MEP pathways. For example, increasing the activity of rate-controlling enzymes in the MVA pathway, while reinforcing metabolic flux through the MEP pathway can expand the intracellular supply of IPP and GGPP (Li et al. 2019; Liao et al. 2016). Additionally, fine-tuning the cofactor biosynthesis pathways and integrating efficient transport mechanisms for precursors and end products can further improve the overall yield of terpenoids in engineered microorganisms (Li et al. 2024c; Liu et al. 2023b).

In conclusion, advancing the understanding and engineering of pathways holds significant promise for optimizing terpenoid production. By leveraging strategies such as gene overexpression, pathway optimization, and cofactor balancing, the biomanufacturing of valuable terpenoid compounds in non-conventional yeast platforms like *Y. lipolytica* can be significantly improved.

### Enhancing the supply of acetyl-CoA

The low yield of terpenoids has been linked to a deficiency in the precursor acetyl-CoA (Nielsen and Keasling 2016). Acetyl-CoA is a crucial central metabolite that plays essential roles in various biosynthetic pathways, including fatty acid synthesis, sterol production, amino acid metabolism, and regulatory processes such as protein and DNA acetylation. Consequently, maintaining sufficient intracellular acetyl-CoA pools is critical for supporting product biosynthesis while preserving metabolic balance within the host cell.

Unlike prokaryotes, the production and transport of acetyl-CoA in yeast are hindered by subcellular compartmentalization. Its synthesis occurs across multiple cellular compartments, including mitochondria, peroxisomes, and cytoplasm (Chen et al. 2012; Wang et al. 2020). Since the majority of natural product biosynthesis pathways are localized in the cytoplasm, the synthesis of cytosolic acetyl-CoA is often a limiting factor in the high production of natural products (Nielsen 2014). Therefore, strategies to increase the cytoplasmic acetyl-CoA pool are crucial for improving diterpenoid yields.

Acetyl-CoA carboxylase (Acc1) catalyzes the ATP-dependent conversion of acetyl-CoA into malonyl-CoA, thereby diverting carbon flux from terpenoid biosynthesis toward fatty acid formation (Tai and Stephanopoulos 2013). The activity of Acc1 is tightly regulated by phosphorylation, which induces conformational changes that reduce its catalytic efficiency. Consequently, relieving this regulatory constraint has emerged as an effective strategy for increasing acetyl-CoA availability and enhancing flux (Hunkeler et al. 2018).

Acetyl-CoA is predominantly concentrated in mitochondria, but it cannot be directly transported across the mitochondrial membrane to the cytoplasm. Because mitochondrial acetyl-CoA is not readily accessible to cytosolic biosynthetic pathways, strategies aimed at increasing its utilization typically involve either relocating key pathway enzymes to mitochondria or enhancing the intracellular trafficking of acetyl-CoA equivalents. The biosynthesis in mitochondria is limited by space, and there is a lack of substrates required for the synthesis of products such as redox potential and cofactors in mitochondria. Acetyl-CoA can be transported from mitochondria to the cytoplasm in the form of citric acid through the mitochondrial citrate alpha ketoglutarate transporter protein (Yhm2). The cytoplasmic citric acid can be converted back into acetyl-CoA by ATP citrate lyase (Acl1 and Acl2) (Castegna et al. 2010; Yuzbasheva et al. 2019). In addition to overexpressing endogenous genes, the heterologous expression of transketolase (Pk) and phosphoacetyltransferase (Pta) can be utilized to construct a two-step phosphoketolase pathway (PK-PTA pathway) to further increase the cytoplasmic acetyl-CoA metabolic flux (Lu et al. 2020). Moreover, peroxisomal carnitine acetyltransferase (Cat2) can also direct acetyl-CoA from peroxisomes to the cytoplasm (Liu et al. 2022). Additionally, in oleaginous yeast *Y. lipolytica*, a large amount of intracellular acetyl-CoA is stored in the form of lipids. Therefore, appropriately weakening lipid synthesis and accelerating fatty acid degradation can regulate the central carbon metabolic flux. Lipids are usually stored in the form of triacylglycerols (TAGs) and are subsequently hydrolyzed by the lipases Tgl3 and Tgl4 to release free fatty acids (Wang et al. 2020). Ma et al. (Ma et al. 2021) overexpressed the key enzymes of the β-oxidation pathway to enhance the lipid degradation pathway and accelerate the decomposition of fatty acids into acetyl-CoA. Blocking certain intracellular pathways of acetyl-CoA consumption can also promote the accumulation of acetyl-CoA. Deletion of the mitochondrial NAD^+^-dependent isocitrate dehydrogenase (IDH1), combined with reinforcement of the upper MVA pathway, redirected carbon flux toward Acl-mediated citrate utilization. This strategy increased cytosolic acetyl-CoA availability and promoted the accumulation of intermediates within the MVA pathway (Rodriguez et al. 2016).

### Blocking the competitive pathway

In the field of terpenoid biosynthesis, various competing metabolic pathways exist, and the deletion or downregulation of these competitive pathways has proven to be an effective strategy for developing high-efficiency cell factories (Pickens et al. 2011). This approach is typically implemented by knocking out genes involved in competitive pathways, reducing their expression using weak promoters, or employing regulated or repressible promoters to enable controllable expression. Notably, the knockout strategy is preferred when the target gene is non-essential to the overall metabolic network.

Minimizing metabolic flux toward competing pathways is also an effective strategy for improving diterpenoid production. Squalene, a major product of the competitive pathway for GGPP, competes with GGPP for the precursor FPP. Because ergosterol is indispensable for maintaining membrane integrity and cellular viability in *S. cerevisiae*, complete disruption of the squalene biosynthetic branch is generally not feasible. Consequently, researchers have focused on modulating *ERG9* transcription through promoter engineering and other regulatory strategies to balance sterol requirements with enhanced terpenoid production (Asadollahi et al. 2007). Given that squalene synthase represents a major competing node for farnesyl diphosphate utilization, modulation of its expression has become a common strategy for improving terpenoid production. Approaches such as promoter replacement with inducible or attenuated promoters and promoter truncation of SQS1 have been used to restrict squalene formation, thereby redirecting precursor flux toward tanshinone biosynthesis and increasing product accumulation. When constructing engineered miltiradiene bacteria, Hu et al. knocked out the activation sequence in the upstream region of the *ERG9* promoter, resulting in an increase in GGPP production from 40.3 mg/L to 196.4 mg/L (Hu et al. 2020). Upc2.1, a constitutively active mutant of the transcription factor Upc2, together with *ERG9* downregulation, increased the availability of GGPP for taxadiene biosynthesis (Engels et al. 2008). By replacing the promoter of the natural *ERG9* gene with MET3, Asadollahi et al. downregulated the expression of *ERG9* gene, weakened the carbon metabolic flow of FPP to ergosterol, and made it flow more to GGPP (Asadollahi et al. 2010).

In addition, in order to reduce by-product and competitive pathway metabolic flow, modifying isoprene catalytic enzymes to enhance GGPP productivity is also an effective means. *ERG20*^F96C^ mutants can continuously catalyse isoprene compounds to produce FPP, and overexpression of *ERG20*^F96C^ in *S. cerevisiae* has become a common strategy to increase GGPP yield (Ignea et al. 2015b). As described in Sect. 3.2, the fusion of *ERG20*^F96C^ with GGPPS not only enhances the spatial proximity of the enzymes but also redirects the metabolic flux from the GPP/FPP pathway (Wei et al. 2022).

### Locating the pathways in subcellular compartments

Unlike prokaryotes, eukaryotic cells possess an extensive network of membrane-bound organelles that enable spatial separation of metabolic activities. This intracellular organization provides dedicated environments for terpenoid biosynthesis and accumulation, with the endoplasmic reticulum (ER), Golgi apparatus, lipid droplets (LDs), peroxisomes, mitochondria, and plasma membrane (PM) serving as important sites for pathway localization and metabolite storage (Delatte et al. 2018).The isolation of the intracellular environment from the cytoplasm provides unique advantages for synthetic biology approaches in yeast (Hammer and Avalos 2017). Targeting biosynthetic pathways to specific organelles offers several advantages for terpenoid production. By physically separating heterologous reactions from competing cytosolic processes, organelle engineering can improve precursor availability and redirect metabolic flux toward the desired products. Furthermore, the localized accumulation of enzymes and intermediates within these compartments promotes more efficient substrate conversion, while the sequestration of hydrophobic metabolites reduces their detrimental effects on cellular physiology (Zhao et al. 2018).

Given that many CYPs and several key enzymes of the MVA pathway are localized to the ER, this organelle provides a favorable platform for the compartmentalized biosynthesis of terpenoids (Hu et al. 2017). Studies have shown that ER expansion can effectively enhance the functional expression of membrane-localized proteins in *S. cerevisiae* (Kim et al. 2019; Liu et al. 2021). ER membrane development is closely linked to cellular phospholipid metabolism, which is modulated by the coordinated action of the transcriptional regulators INO2, INO4, and OPI1 (Gardenour et al. 2004). In addition to constructing artificial CYP-CPR fusion proteins to enhance enzymatic catalysis, the three CYPs involved in forskolin biosynthesis are localized to the ER membrane. Increasing ER membrane abundance through INO2 overexpression improved the expression of CYP76AH enzymes and contributed to higher product titers (Ju et al. 2022). Likewise, deletion of PAH1 induced extensive ER proliferation in both *S. cerevisiae* and *Y. lipolytica*, further demonstrating the value of ER engineering for supporting CYP-mediated diterpenoid biosynthesis (Arendt et al. 2017; Guerfal et al. 2013).

Peroxisomes are one of the main sites for fatty acid β-oxidation, a process that generates large amounts of acetyl-CoA. Additionally, peroxisomes contain a NADPH/NADP^+^ pool, which is crucial for the catalytic reactions of many diterpenoid synthases, such as CYPs, that require substantial NADPH as a reducing power (Okumoto et al. 2020). Therefore, targeting terpenoid synthesis to the peroxisome and reconstructing the MVA pathway within this organelle can enable efficient oxidative modifications (Dusseaux et al. 2020). While this study primarily focuses on the synthesis of other terpenoid compounds, the approach is also highly relevant for the reconstruction of diterpenoid oxidative modification pathways within peroxisomes. Peroxisomes have emerged as versatile organelles for both the biosynthesis and sequestration of terpenoids, particularly in non-oleaginous yeast hosts. Moreover, their abundance and morphology can be engineered through the modulation of peroxisome biogenesis and proliferation pathways, providing a flexible platform for enhancing terpenoid production (Choi et al. 2022).

The biosynthesis of terpenoids is mainly carried out in mitochondria, which provide abundant acetyl-coA and other substrates (Duran et al. 2020). The benefits of mitochondrial compartmentalization for enhancing biosynthetic pathway performance were clearly demonstrated by Zhu et al. (Zhu et al. 2021). Because mitochondria occupy a relatively small proportion of the total cellular volume, Hasunuma et al. expanded mitochondrial size by disrupting genes involved in mitochondrial morphology. Coupling this strategy with the introduction of the MVA pathway into mitochondria significantly enhanced the production of IPP and DMAPP (Yanagibashi et al. 2024).

LDs consist of a neutral lipid core surrounded by a phospholipid monolayer decorated with proteins involved in lipid metabolism and organelle homeostasis. The frequent localization of terpenoids within LDs suggests that these organelles serve as important intracellular reservoirs for hydrophobic metabolites. This natural storage capacity has attracted considerable interest in synthetic biology, as engineering LD biogenesis and dynamics offers an attractive target to enhance both the accumulation and storage of terpenoids in microbial hosts (Arhar and Natter 2019).

### Engineering the metabolism through dynamic control

In the past, during the construction of microbial cell factories, efforts to enhance product synthesis pathways or reduce carbon metabolism diversion often involved overexpressing target genes (or pathways) or blocking competitive pathways (Jiang et al. 2017; Xie et al. 2015; Yuan and Ching 2015). However, the engineered strains are sensitive to environmental fluctuations, and their production performance is prone to decline during large-scale fermentation (Liu et al. 2018a, ). In addition, static regulatory strategies may lead to cofactor imbalances, thereby compromising overall productivity. Thus, research has focused on how to employ appropriate metabolic regulation methods at the right time to modulate gene expression levels. The application of dynamic regulation strategies has proven to be an effective approach for boosting the production capacity of cell factories. This strategy closely resembles the natural regulatory mechanisms in microorganisms, which adjust cellular metabolic status in real time by sensing environmental and intracellular signals, thereby allocating carbon and energy within cells in a more moderate and conservative manner (Shen et al. 2019).

Currently, various dynamic control elements based on metabolite responses have been developed to achieve the dynamic distribution of substance and energy flow in metabolic networks. These elements reduce the impact of heterologous pathways on cell growth and the balance of metabolic flux, while effectively regulating the efficient synthesis of various high-value-added products (Ghodasara and Voigt 2017; Portela et al. 2017). The development of metabolite-responsive biosensors has enabled the integration of cellular sensing and pathway regulation. Such systems can detect key metabolic intermediates and trigger corresponding genetic responses, facilitating dynamic optimization of engineered biosynthetic processes (Dekker and Polizzi 2017). For instance, transcription factors responsive to diterpenoid precursors can be harnessed for dynamic metabolic regulation. In yeast, engineered FPP-sensing transcription factors enable feedback control in which FPP accumulation downregulates the expression of upstream genes in the MVA pathway, such as *HMG1*, thereby preventing excessive precursor buildup (Cao et al. 2023).

Quorum sensing (QS) regulates gene expression in response to threshold concentrations of signaling molecules (Portela et al. 2017; Tsao et al. 2010; Zargar et al. 2015). In the LuxI/LuxR quorum-sensing system of *E. coli*, LuxI produces N-acyl homoserine lactone (AHL) signals that activate LuxR-dependent transcription once sufficient signal molecules accumulate in the extracellular environment (Kim et al. 2017). A similar principle operates in *Pseudomonas aeruginosa*, where a multilayered QS regulatory network—comprising the LasI/LasR, RhlI/RhlR, and PQS systems—coordinates virulence factor expression and biofilm formation through synergistic interactions (Liang et al. 2012; Wilder et al. 2011).

In engineered microbial hosts, diterpenoid synthase genes can be placed under the control of QS-responsive promoters, enabling an autonomous transition from a growth phase to a production phase without the need for external inducers. This self-regulated growth-to-production switch reduces process complexity and lowers cultivation costs.

## Research needs and future directions

Diterpenoids constitute a diverse family of natural products with complex structures and remarkable biochemical diversity. Recent advances in metabolic engineering and synthetic biology have significantly expanded the capability of microbial cell factories to produce these complex molecules, providing a sustainable alternative to traditional plant-based production (Bureau et al. 2023). To date, microbial modification has enabled the efficient synthesis of various diterpenoid compounds, such as cis-abienol (Li et al. 2019; Zhang et al. 2022), miltiradiene (Hu et al. 2020), sclareol (Cao et al. 2023; Einhaus et al. 2022; Sun et al. 2024; Wu et al. 2025), ent-kaurane (Chen et al. 2024; Geiselman et al. 2020), etc. However, the titers of many other diterpenoids, including gibberellin, carnosic acid, and forskolin, remain relatively low (Table 1). The construction of highly efficient microbial platforms for terpenoid production remains an ongoing endeavor. Key challenges include optimizing pathway performance, improving host robustness, and achieving economically viable production at industrial scale.

Currently, high-yield production of target products is typically achieved through the modification of chassis microorganisms, including enhancing the MEP/MVA pathway, optimizing the supply of cofactors (such as NADPH/ATP), and reducing competitive pathways (Paramasivan and Mutturi 2017; Zhou et al. 2012). In addition, the challenge of heterologous expression of diterpenoid synthases is often mitigated through strategies such as enzyme engineering for constructing fusion tags and co-expression of chaperones (Xu et al. 2023). Ultimately, dynamic regulation (e.g., promoter engineering, CRISPRi) to balance the requirements between growth and synthesis phases can further enhance the production of diterpenoids (Lauchli et al. 2013).

With advancements in synthetic biology, genomics, and metabolomics technologies, progress has been made in the heterologous synthesis of diterpenoids by microorganisms. Nevertheless, new strategies are still needed to address the emerging challenges. First, more advanced synthetic biology tools are required to further improve diterpenoid production. In the near term, multi-omics approaches, including transcriptomics, proteomics, metabolomics, and fluxomics, will play an essential role in identifying metabolic bottlenecks, uncovering regulatory targets, and guiding rational pathway optimization in microbial hosts (Lee et al. 2012; Nielsen and Keasling 2016). In addition, computational tools and machine learning-assisted approaches are increasingly being applied to enzyme discovery, protein engineering, and pathway design. These methods can accelerate the identification of promising heterologous enzymes and facilitate the optimization of metabolic networks. Looking further ahead, the convergence of artificial intelligence and synthetic biology may enable automated design-build-test-learn (DBTL) cycles, predictive modeling of fermentation processes, and dynamic control of metabolic pathways, thereby accelerating the development of highly efficient microbial cell factories (Hafner et al. 2021).

Second, exploring new enzymes and alternative pathways remains crucial for advancing diterpenoid biosynthesis. In addition to discovering uncharacterized diTPSs that generate novel carbon skeletons, the identification of enzymes with superior catalytic efficiency, substrate specificity, and expression characteristics is of greater practical importance for industrial production. Recent advances in genome mining, phylogenetic analysis, motif-guided screening, and functional characterization have significantly accelerated the discovery of TPSs from diverse organisms (Huang et al. 2021). These approaches not only expand the repertoire of diterpenoid biosynthetic enzymes but also provide valuable biocatalysts for improving production efficiency. (Li et al. 2023; Yang et al. 2017).

Furthermore, the development of alternative precursor-supplying pathways offers new opportunities to overcome limitations associated with conventional MVA and MEP pathways. A representative example is the isopentenol utilization pathway (IUP), which converts exogenously supplied isoprenol and prenol into IPP and DMAPP through only two enzymatic steps (Chatzivasileiou et al. 2019). Compared with native isoprenoid pathways, the IUP pathway features a shorter route, reduced metabolic burden, and improved carbon conversion efficiency. The integration of such alternative pathways with traditional metabolic engineering strategies may provide an effective approach for enhancing precursor availability and increasing diterpenoid titers in microbial hosts. The continued discovery of efficient enzymes and innovative biosynthetic routes will facilitate the production of structurally diverse diterpenoids and accelerate their industrial application.

Finally, developing scalable bioprocesses based on engineered microorganisms is a core direction in the current field of biotechnology (Liu et al. 2023b). Large-scale fermentation processes can achieve stable scale-up from laboratory (milliliter level) to industrial (cubic meter level) production, reducing unit production costs. Designing culture media suitable for the growth of engineered microorganisms and diterpenoid synthesis can improve substrate utilization and product yields (Einhaus et al. 2022). Additionally, two-phase culture systems reduce product feedback inhibition by adding appropriate organic phases (such as oleic acid or resin) to adsorb hydrophobic diterpenoids (Chen et al. 2025). Furthermore, developing efficient methods for product separation and purification from large-volume fermentation broths is critical for reducing production costs, improving product recovery, and facilitating the industrial implementation of microbial diterpenoid production.

Although many of the engineering strategies discussed in this review, such as precursor pathway optimization, enzyme engineering, compartmentalization, and dynamic regulation, are broadly applicable to terpenoid biosynthesis, diterpenoids present several unique challenges that warrant dedicated investigation. Compared with mono-, sesqui-, and most triterpenoids, diterpenoids generally possess larger and more structurally complex carbon skeletons, which often require multiple downstream tailoring reactions catalyzed by CYPs, reductases, and GTs. The functional expression and coordination of these enzymes frequently become major bottlenecks in microbial hosts. In addition, many diterpenoids exhibit high hydrophobicity and poor aqueous solubility, leading to intracellular accumulation, product toxicity, and secretion limitations. These characteristics may necessitate specialized engineering strategies, including tailored transporter engineering, subcellular compartmentalization, and improved product sequestration systems. Currently, most metabolic engineering approaches employed for diterpenoid production are adapted from general terpenoid engineering frameworks, and truly diterpenoid-specific strategies remain limited. Therefore, the development of engineering approaches specifically targeting the unique biochemical and physicochemical properties of diterpenoids represents an important direction for future research.

## Data Availability

Not applicable.
